# Invasive Fire Ants Reduce Reproductive Success and Alter the Reproductive Strategies of a Native Vertebrate Insectivore

**DOI:** 10.1371/journal.pone.0022578

**Published:** 2011-07-20

**Authors:** Russell A. Ligon, Lynn Siefferman, Geoffrey E. Hill

**Affiliations:** 1 Department of Biological Sciences, Auburn University, Auburn, Alabama, United States of America; 2 Department of Biological Sciences, Appalachian State University, Boone, North Carolina, United States of America; 3 Department of Biological Sciences, Auburn University, Auburn, Alabama, United States of America; University of Veterinary Medicine Hanover, Germany

## Abstract

**Background:**

Introduced organisms can alter ecosystems by disrupting natural ecological relationships. For example, red imported fire ants (*Solenopsis invicta*) have disrupted native arthropod communities throughout much of their introduced range. By competing for many of the same food resources as insectivorous vertebrates, fire ants also have the potential to disrupt vertebrate communities.

**Methodology/Principal Findings:**

To explore the effects of fire ants on a native insectivorous vertebrate, we compared the reproductive success and strategies of eastern bluebirds (*Sialia sialis*) inhabiting territories with different abundances of fire ants. We also created experimental dyads of adjacent territories comprised of one territory with artificially reduced fire ant abundance (treated) and one territory that was unmanipulated (control). We found that more bluebird young fledged from treated territories than from adjacent control territories. Fire ant abundance also explained significant variation in two measures of reproductive success across the study population: number of fledglings and hatching success of second clutches. Furthermore, the likelihood of bluebird parents re-nesting in the same territory was negatively influenced by the abundance of foraging fire ants, and parents nesting in territories with experimentally reduced abundances of fire ants produced male-biased broods relative to pairs in adjacent control territories.

**Conclusions/Significance:**

Introduced fire ants altered both the reproductive success (number of fledglings, hatching success) and strategies (decision to renest, offspring sex-ratio) of eastern bluebirds. These results illustrate the negative effects that invasive species can have on native biota, including species from taxonomically distant groups.

## Introduction

Since Darwin [1], ecologists have often assumed that competition for food resources is most intense among closely related species [2]. Feeding guilds, however, often include a diverse assemblage of taxa competing for the same resources [3]–[5]. For example, in desert habitats of the American Southwest, ants, diurnal birds, and nocturnal mammals all compete for the same seeds (reviewed in [6]), and the abundance of each species in the guild affects the abundance of the others.

Ants and birds are among the most abundant consumers in many ecosystems [7], [8] and they frequently compete for resources. Competitive relationships between ants and birds can influence the distribution [9], habitat-use [10], foraging behavior [11], [12], and offspring quality [13] of birds. Although ants can impact bird behavior via interference competition, wherein ants actively exclude avian competitors from a given area or resource [11], indirect competition for common food resources appears to underlie most competitive interactions between ants and birds. The change in availability and distribution of nutritional resources brought about by foraging ants can influence the distribution and behavioral decisions of birds such that they avoid settling in areas with high ant densities [9] and avoid specific trees containing high densities of foraging ants [12]. In addition to modifying the behaviors of birds, ants can also reduce the reproductive success of birds [14].

To date, studies exploring the dynamics of ant/bird interactions have focused primarily on species that naturally co-occur (but see [15]). Given the negative impacts that native ants can have on native birds, and the large impacts that invasive species can have on native flora and fauna [16], we were curious about the competitive effects that an introduced ant (red imported fire ants, *Solenopsis invicta*) might have on a native insectivorous bird (eastern bluebirds, *Sialia sialis*). Eastern bluebirds are obligate cavity-nesting, double- or triple-brooded passerine birds common throughout eastern North America [17]. Red imported fire ants were introduced into the United States from South America in the 1930's [18] and have subsequently become a serious ecological problem in much of the southern U.S. [19]. Fire ants disrupt natural food webs [19] and negatively impact native flora [20] and fauna [21], [22], frequently devastating local arthropod abundance and diversity [21] (but see [23] for an evaluation of long-term arthropod community recovery).

Because fire ants can dramatically alter arthropod communities [21], their abundance could indirectly influence the foraging and reproductive success of insectivorous animals. Indeed, experimental reduction of fire ant abundance has been shown to increase the abundance of the insectivorous loggerhead shrike (*Lanius ludovicianus*; [24]). To our knowledge, the study by Allen et al. [24] is the only published experimental study of the competitive effects that fire ant invasions have on insectivorous birds. However, a link between fire ant invasion and reduced reproductive output has previously been suggested for eastern bluebirds (P. Gowaty, unpublished data). Although fire ants occasionally prey on bluebird nestlings, the impact of this invasive ant species on bluebirds due to competition for food is predicted to be substantially greater than losses caused by occasional nest predation [17].

To further examine the impact of invasive fire ants on a native insectivorous bird species, we measured the effects of fire ant abundance on the breeding biology of eastern bluebirds by use of both correlational and experimental methods. First, we examined whether variation in foraging fire ant abundance across bluebird territories was correlated with various parameters of reproductive success (number of fledglings, hatching success) and reproductive strategy (likelihood of producing a second brood in the same territory (re-nesting), offspring sex ratio). Second, we compared the reproductive success and reproductive strategies of bluebirds inhabiting territories with experimentally reduced fire ant abundances to those inhabiting adjacent territories with high fire ant densities. We predicted that resource competition between ants and bluebirds would lead to reduced reproductive success and altered reproductive strategies in bluebird territories with abundant fire ants. Specifically, we predicted that the depletion of arthropod food resources by fire ants would decrease habitat quality in the territories they occupied and cause bluebirds inhabiting such ant-abundant territories to produce female-biased broods [25].

## Methods

### Ethics Statement

This research was approved by the Auburn University Internal Animal Care and Use Committee (IACUC project registration no. 2008-1341) and conducted under Alabama State and U.S. Fish and Wildlife permits.

### Study Species

We examined the effects of fire ant abundance on a banded population of eastern bluebirds in Lee County, Alabama, USA during the spring and summer of 2009. Eastern bluebirds are an insectivorous passerine species that readily use man-made nest boxes for their nests [17]. Nest sites are typically at the center of the all-purpose territories (defined as territories for “display, courtship, paternity, nest seclusion, and feeding”; [26]) that bluebirds defend during the breeding season (late March-August; [17]). We monitored bluebird nest boxes that were placed primarily in livestock pastures, fields next to roads and ponds, and in pastoral habitat near the Auburn University campus.

### Study Design

Because differences in habitat quality and local resource abundance are likely to affect the reproductive success and strategies of bluebirds inhabiting different territories, we used a paired experimental design incorporating dyads of territories selected for their proximity and habitat homogeneity. Territory dyads were comprised of two adjacent territories, with each territory centered on a single nest box. Within each territory dyad, nest boxes in adjacent territories were approximately 150 m apart and were located in similar habitat types (e.g., both in open fields, both adjacent to a road, both near forest edge, etc). The 150 m spacing was close enough to ensure that habitat parameters were likely similar, but far enough apart that the application of fire ant poison affected only the treated territory. Prior to the 2009 breeding season we selected 23 such territory pairs, with each territory centered on a nest box used by bluebirds in previous years with one exception. One territory dyad made up of two new nest boxes was also incorporated into the study.

### Bluebird Measurements

We monitored 71 nest boxes (46 paired territories and 25 unpaired territories) through the breeding season to determine when nests were in use by bluebirds, to monitor the number of eggs laid and hatched, and to record the age and development of each bluebird offspring. When nestlings were 14 days old, we gathered feather samples from each nestling and recorded sex based on feather coloration. In well-developed nestlings, researchers can reliably estimate offspring sex based upon the amount of blue in emerging flight feathers [27], [28]. Additionally, adult bluebirds were captured and color banded for field identification. Many nest boxes were used by bluebird pairs for up to three nesting attempts during the breeding season, but we focused our analyses only on the first two breeding attempts at each nest box.

Although we reduced snake predation upon eggs and nestlings by placing corrugated tin guards around the poles supporting nest boxes, snakes still depredated some nests. Predation events, causing the failure of entire nests, occurred throughout the course of the breeding season but showed a marked increase later in the season. Predation influenced not only the number of offspring fledged from a given nest, but also the energetic demands faced by the parents. Because predation so dramatically skewed reproductive parameters, and did so irrespective of parental effort or food resources, we excluded nests subjected to predation from our final analyses. Although snakes destroyed some nests, axle grease applied to the nest box posts prevented the loss of any nests due to fire ant predation.

### Fire Ant Treatment and Measurement

To assess the impact of fire ants on the reproductive success and strategies of bluebirds, fire ant populations were suppressed on one randomly chosen territory from each dyad using commercially available, ant-specific hydramethylnon bait (Amdro, American Cyanamid, Wayne, NJ). This type of bait provides an ideal means to control fire ants in experimentally treated plots without greatly impacting other arthropods. Amdro fire ant bait is composed of an inert, corn grit carrier infused with soybean oil, which is attractive to foraging fire ants but is typically ignored by other insects [29]. Foraging fire ants find the bait and take it back to the colony, wherein the toxicant is spread throughout the mound, affecting all members of the colony. Using hand-operated dispensers, we spread fire ant bait over roughly 5024 m^2^ in each treated territory. This area represents approximately 25% of the average documented eastern bluebird territory (2.1 ha) [17], [30]. For each treated plot, we applied bait to all suitable habitat for fire ants, incidentally the same type of habitat favored for foraging by bluebirds, within a 40-m radius of the nest box in that territory. Territories were treated once between late March and early April, before most bluebirds at our study site had begun to breed, but after males had settled and were defending territories. The timing of our bait application eliminated the likelihood that any observed differences in the reproductive success or strategies of bluebirds in treated and untreated plots arose as a result of preferential settlement by superior individuals on treated territories.

To assess the effectiveness of our methods for fire ant reduction, as well as to measure variation in fire ant abundance, we monitored the foraging abundance of fire ants by trapping foraging ants in baited plastic vials [31]. Within each territory, we placed eight vials containing 2.5 cm sections of hot dog (processed meat) on the ground and shaded the vials with opaque plastic plates mounted on 16-gauge wire. Vials were placed 20 m from the nest box at the four cardinal and four intermediate directions (15.3 m apart) when suitable habitat (excluding roads and water) existed at these locations. Although the spatial characteristics of several territories (e.g. those next to ponds) prevented us from deploying all eight baited vials, we attempted to deploy the maximum number of vials, spaced accordingly, in suitable fire ant habitat. After 30 min vials were retrieved, capped, and frozen. After being trained by a local fire ant expert (LC Graham, Auburn University) and consulting an illustrated key [32], we identified and counted the frozen ants. Interspecific competition for the hot dog baits was very low (only 4% of vials contained ant species other than *S. invicta*) and appeared roughly equivalent in treated and untreated territories (non-fire ant species were captured in 5 control territories and 5 treated territories). To account for the variation in number of vials deployed, we quantified relative fire ant abundance on each territory by dividing the total number of fire ants captured at that territory by the number of vials deployed. We measured fire ant abundance when nestling bluebirds were 5–12 days old.

### Analyses

To examine the potential effects of fire ants on bluebirds, we conducted several different analyses. First, we used paired t-tests, wherein the control and treated territory within each dyad represented the paired unit of analysis. We analyzed a) the effect of our hydramethylnon treatment on foraging fire ant abundance, b) whether the number of fledglings differed between control and treated territories (first and second nests analyzed separately to test for temporal effects, then pooled to test for season-long effects), and c) whether the offspring sex ratio was different between control and treated territories. Second, we used chi-square analyses to compare a) the likelihood of nest predation between control and treated territories and b) the likelihood of parents re-nesting in the same territory where they raised their first brood. Third, we used linear regression to determine whether variation in fire ant abundance could predict variation in the number of fledglings produced. Fourth, we used logistic regression to determine whether variation in fire ants could predict likelihood of re-nesting. Fifth, we used generalized linear models (PROC GENMOD in SAS) with binomial error distributions and logit link functions to determine whether fire ant abundance could predict hatching success across our bluebird population. Generalized linear models are appropriate for binary and proportional data, and can incorporate both continuous and categorical explanatory variables [33].

## Results

During the initial wave of nesting attempts by eastern bluebirds at our study site in 2009, bluebirds nested in both control and treated territories in 19 of the 23 dyads of adjacent territories. Of these 19 territory dyads, both members of 15 dyads experienced no predation events. Within this subset of predation-free territory pairs, 11 of 15 nest boxes in control territories were used during the second nesting attempt of the season and 10 of 15 nest boxes in treated territories were re-used (though not necessarily by the same pair).

### Fire Ant Treatment

The abundance of foraging fire ants, measured as the mean number of ants per collection vial, was significantly lower in territories treated with the hydramethylnon ant bait (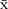 = 38.9 ± SE 11.8, range 0.0–142.2) compared to adjacent control territories (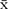 = 128.8±27.9, range 6.67–352.25; paired t-test, *n* = 15, *t* = 3.2, *p* = 0.006).

### Predation

Nest predation was equally likely in treated and untreated territories (overall probability of predation  =  0.13, *X* ^2^ = 0.56, d.f. = 1, *p* = 0.45). We also compared nest predation across all territories to see if fire ant abundance was related to the likelihood of nests being preyed upon. We found no relationship between foraging ant abundance and predation in first nests (*n* = 38, *p* = 0.66), second nests (*n* = 31, *p* = 0.44), or all nests combined (*n* = 69, *p* = 0.62).

### Fledged Offspring

Bluebird pairs inhabiting treated territories did not fledge significantly more offspring (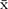 = 3.9±0.4) than pairs in control territories (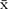 = 3.0±0.4) during the first breeding attempt of the season (paired t-test, *n* = 15, *t* = 1.60, *p* = 0.13). Additionally, there was no significant difference between the number of young fledged by bluebirds in treated (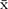 = 3.6±0.3) or control (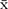 = 3.4±0.6) territories during second nesting attempts (paired t-test, *n* = 7, *t* = 0.31, *p* = 0.77). However, within dyads, the cumulative fledging success (total number of offspring fledged from a given territory) was significantly different between control and experimental territories. Specifically, when we analyzed the total number of offspring fledged from each territory (regardless of parent identity and excluding nests that experienced predation), we found that treated territories fledged 1.1 more offspring (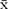 = 6.3±0.5) than adjacent control territories (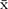 = 5.2±0.5; paired t-test, *n* = 15, *t* = 2.17, *p*<0.05). This last statistic compares the productivity of territories rather than individuals and takes into account the territories where no second nest was attempted (4/15 control territories and 5/15 treated territories).

**Figure 1:**
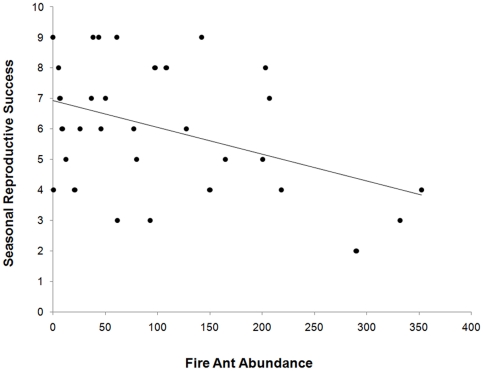
Seasonal reproductive success of bluebirds as a function of fire ant abundance. Relationship between fire ant abundance (mean number of ants captured per sample vial) and the seasonal reproductive success (total number of fledged offspring) of eastern bluebirds (*R* ^2^ = 0.17, *F*_1, 31_ = 5.98, *p* = 0.02). Each point represents a single territory (n = 31).

In addition to comparisons of territory dyads, we compared total fledging success across all territories (including unpaired territories) relative to fire ant abundance. Among nest boxes that were never depredated (in either first or second nesting attempts), we found that increased fire ant abundance was associated with decreased reproductive output (*R* ^2^ = 0.17, *F*_1, 31_ = 5.98, *p* = 0.02; Fig. 1).

### Hatching Success

**Figure 2:**
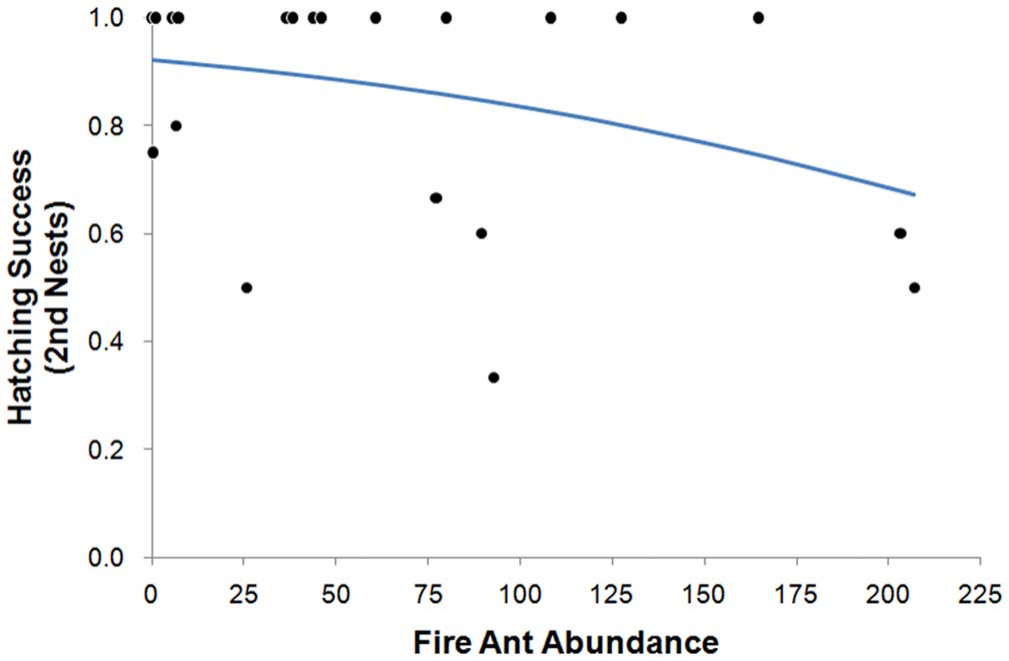
Hatching success of bluebirds as a function of fire ant abundance. Relationship between fire ant abundance and hatching success of second nests of eastern bluebirds (*n* = 21). Logistic regression equation (Y = e^2.4788 -0.0085(fire ant abundance)^/1+ e^2.4788 - 0.0085(fire ant abundance)^), where Y is equal to the hatching success, represented by the line.

We found that hatching success was not related to fire ant abundance during first broods (*n* = 32, *p* = 0.40) but that the hatching success of mothers who re-nested in their original territories (and for whom we had fire ant data, *n* = 21) was negatively related to the abundance of foraging fire ants (*p* = 0.051, Fig. 2).

### Offspring Sex-Ratio

We were able to assess the sex of every nestling from 13 territory dyads (26 nests) during the first breeding attempts of the season. Broods in treated territories had, on average, 22% more males than broods in adjacent control territories (average proportion of males in treated territories  =  0.71±0.06, average proportion of males in control territories  =  0.49±0.10; paired t-test, *n* = 13, *t* = 2.63, *p* = 0.02). In second nests, broods in treated territories contained 22% more males than broods in adjacent control territories (average proportion of males in treated territories  =  0.62±0.14, average proportion of males in control territories  =  0.40±0.14). Despite the apparent disparity, the differences between the two groups were not significant for second nests (paired t-test, *n* = 5, *t* = 1.04, *p* = 0.36), likely owing to small sample size.

### Likelihood of Re-Nesting

**Figure 3:**
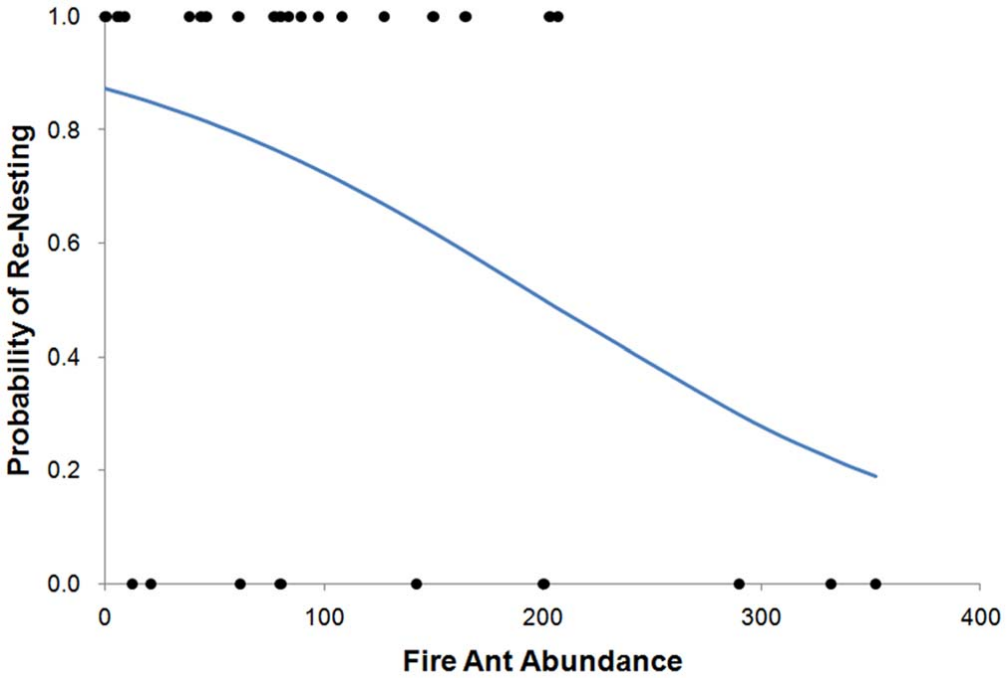
Probability of bluebirds re-nesting in their original territory as a function of fire ant abundance. Relationship between fire ant abundance (mean number of ants captured per sample vial) and the calculated probability of re-nesting by the same eastern bluebird parents (*n* = 29). Logistic regression equation (Y = e^1.9204 -0.00958(fire ant abundance)^/1+ e^1.9204 - 0.00958(fire ant abundance)^), where Y is equal to the probability of re-nesting, represented by the line.

To determine whether bluebird parents re-used the same nest box during their second breeding attempt of the season we had to capture both parents at the nest box during both breeding attempts. After analyzing the 39 nest locations across the study population where either a) we positively identified mothers and fathers from both first and second broods or b) no second nesting attempt occurred, we determined that re-nesting by one or both of the original parents at a nest was equally likely in both treated and untreated territories (overall probability of re-nesting  =  0.64, chi-square between control and treated territories  =  0.03, d.f. = 1, *p* = 0.86). However, when we used a logistic regression to analyze the likelihood of re-nesting across territories for which we had data on abundance of foraging fire ant and re-nesting information (*n* = 29 nests), we found a significant negative relationship between fire ant abundance and likelihood of re-nesting (*n* = 29, *p* = 0.045). This effect may be driven by territories with very high fire ant numbers and the fact that parents at these nests did not undertake a second breeding attempt at the same site (Fig. 3).

## Discussion

The abundance of foraging fire ants had a marked effect on the breeding success and reproductive strategies of eastern bluebirds. Bluebirds that nested in territories with abundant fire ants produced fewer fledglings, had more hatching failures during second broods, produced fewer male offspring, and were less likely to re-nest in their original territories than neighboring bluebirds on territories with fewer ants. These results confirm the importance of cross-phyletic competition in shaping ecological community dynamics, and demonstrate the strong impact that invasive species can have on native animals.

The effects of fire ants on the reproductive success of bluebirds were not significant during either the first or second nesting attempts when each was considered alone, although the trends during each reproductive bout were in the predicted directions with treated territories producing more offspring than control territories. There was, however, a significant difference between treated and control territories in total reproductive output (number of fledged offspring) over the course of the entire breeding season. These observations are similar to those reported by Aho et al. [14], who found that differences in reproductive output between Eurasian treecreepers (*Certhia familiaris*) inhabiting territories with experimentally manipulated levels of red wood ants (*Formica rufa*) were most pronounced when treecreepers were double rather than single-brooded. For both treecreepers and bluebirds, prolonged competition with ants appears to take a gradual toll, which becomes most evident after repeated bouts of reproduction.

The mechanisms by which greater fire ant abundance reduces reproductive success in bluebirds are not entirely clear. Likely, ants affect bluebirds primarily by competing for arthropod food, but there are at least two mechanisms by which lower food resources could impact reproductive success. First, less food on a territory may subject parents to additional stress, which could shift time budgets towards self-maintenance behaviors and away from offspring care [34]. Second, reduced food availability could increase the distance that bluebird parents must travel to provide sufficient resources to their offspring (*sensu* [35]), increasing the work that parents must perform to successfully raise offspring. Previously, it has been shown that higher workloads, in the form of experimentally enlarged broods, reduce the likelihood of female bluebirds producing second clutches [36]. If providing food to many offspring (as in [36]) and providing adequate food when resources are scarce put similar strains on bluebird parents, both stressors could result in decreased seasonal reproductive output.

Because fire ants are known to disrupt the native arthropod communities [21] upon which bluebirds rely for food, and because food abundance is known to influence hatching success in a number of birds (e.g. carrion crows *Corvus corone*, [37]; white storks *Ciconia ciconia*, [38]; black-legged kittiwakes *Rissa tridactyla*, [39]), we predicted that a reduced availability of food for parents in territories with high fire ant densities would depress hatching success. However, we found reduced hatching success among broods in territories with high densities of fire ants only during second breeding attempts. The reason we failed to detect differences in hatching success in the first nests of the season but did detect differences in second nests is likely a result of cumulative effects of insufficient food on the condition of breeding bluebirds. Consequently, significant negative effects on hatching success may only manifest after prolonged exposure to fire ant-mediated food stress [40]. Alternatively, it is possible that the arthropod communities on which bluebirds depend did not have time to respond to the experimental reduction of fire ants by the time of the first nesting attempt. Because the success of individual bluebird nests was typically an all-or-none response, with respect to hatched nestlings, the reduction in hatching rate is likely largely responsible for the decreased fledgling output of control territories.

Fire ants not only reduced the reproductive output of bluebirds and reduced hatching success; they also triggered a shift in the sex ratio of bluebird offspring. Although the mechanisms of primary sex-allocation are currently unknown for birds [41], numerous studies support the idea that females can alter the sex-ratio of their offspring and do so in response to both social [42], [43] and environmental [25], [44] factors. Because reproductive variance is usually greater for males than females [45], [46], and because high-quality males will produce more offspring than high-quality females, sex ratio theory suggests that parents should increase investment in male offspring when conditions are favorable for producing high-quality offspring [47], [48]. In concordance with these predictions, bluebird clutches from territories with experimentally reduced fire ant abundance had, on average, 22% more males than clutches in adjacent territories. Although this difference was statistically significant only for nests in the first half of the breeding season, our sample size during second nesting attempts severely limited our statistical power to detect real differences. It is interesting to note that the significant differences in sex ratio between adjacent territories in first nests arose not from the reduction of male offspring in habitats with more fire ants (average proportion of males  =  0.49), but from an increase in the proportion of male offspring in territories with fewer ants (average proportion of males  =  0.71). The direction of this response indicates that an experimental reduction of fire ants improves local resources and maternal condition, both of which should favor the increased production of male offspring.

Invasive species can occasionally exert selection pressure strong enough to induce rapid evolutionary changes in native biota (e.g. [49], [50]). With respect to fire ants, any adaptive responses by bluebirds are likely to include changes in habitat and selection of breeding sites. To avoid the possibility that bluebird used post-manipulation fire ant abundance as a criterion in choosing territories, we applied the hydramethylnon fire ant bait only after bluebirds had already chosen territories. Although our treatment regime precluded the possibility that bluebirds chose territories based on experimentally manipulated levels of fire ants, it did not prevent bluebirds from relocating after their first breeding attempt. Re-nesting by one or both parents, however, was equally likely (∼64%) in both treated and control territories. Nevertheless, the probability that bluebirds would stay in their original territories was negatively correlated with the fire ant abundance in that territory. This relationship appears to be driven by the fact that bluebirds in territories with high abundance of foraging fire ants (>250 ants/vial) did not re-nest. Relocation may be a simple behavioral strategy in response to reduced food abundance in territories with large numbers of fire ants, rather than an active assessment of fire ant abundance.

The primary insight from this study is not the negative effect of invasive fire ants on the abundance of eastern bluebirds, which is a common songbird throughout their range and not a species of conservation concern [17]. Rather, the more important implication of our study is that invasive fire ants can be a significant competitor of insectivorous birds, and perhaps other insectivorous vertebrates, and can reduce the reproductive success of such species. Several other insectivorous birds that nest in pasture and farmland in the southeastern United States where fire ants have been introduced, including the eastern meadowlark (*Sturnella magna*), northern mockingbird (*Mimus polyglottus*), and brown thrasher (*Toxostoma rufum*), have shown persistent declines in recent decades [51]–[53]. The declines of these species are often attributed to habitat alteration but, based on the observations from our study, competition with invasive fire ants should be included among the factors that might be contributing to the decline of these insectivorous bird species. Given the predicted global expansion of fire ants [54], understanding the detrimental effects that fire ants may have on insectivorous birds could become even more important in the future.
